# MDA5 cleavage by the Leader protease of foot-and-mouth disease virus reveals its pleiotropic effect against the host antiviral response

**DOI:** 10.1038/s41419-020-02931-x

**Published:** 2020-09-02

**Authors:** Miguel Rodríguez Pulido, Encarnación Martínez-Salas, Francisco Sobrino, Margarita Sáiz

**Affiliations:** Centro de Biología Molecular Severo Ochoa, CSIC-UAM, Madrid, Spain

**Keywords:** Mechanisms of disease, RIG-I-like receptors

## Abstract

The RIG-I-like receptor (RLR) melanoma differentiation-associated gene 5 (MDA5) plays a key role in triggering innate antiviral response during infection by RNA viruses. MDA5 activation leads to transcription induction of type-I interferon (IFN) and proinflammatory cytokines. MDA5 has also been associated with autoimmune and autoinflammatory diseases by dysfunctional activation of innate immune response in the absence of infection. Here, we show how foot-and-mouth disease virus (FMDV) counteracts the specific antiviral effect exerted by MDA5 targeting the protein for cleavage by the viral Leader protease (Lpro). MDA5 overexpression had an inhibitory effect on FMDV infection in IFN-competent cells. Remarkably, immunostimulatory viral RNA co-immunoprecipitated with MDA5 in infected cells. Moreover, specific cleavage of MDA5 by Lpro was detected in co-transfected cells, as well as during the course of FMDV infection. A significant reduction in IFN induction associated with MDA5 cleavage was detected by comparison with a non-cleavable MDA5 mutant protein with preserved antiviral activity. The Lpro cleavage site in MDA5 was identified as the RGRAR sequence in the conserved helicase motif VI, coinciding with that recently reported for Lpro in LGP2, another member of the RLRs family involved in antiviral defenses. Interestingly, specific mutations within the MDA5 Lpro target sequence have been associated with immune disease in mice and humans. Our results reveal a pleiotropic strategy for immune evasion based on a viral protease targeting phylogenetically conserved domains of immune sensors. Identification of viral strategies aimed to disrupt MDA5 functionality may also contribute to develop new treatment tools for MDA5-related disorders.

## Introduction

The retinoic acid-inducible gene-I (RIG-I)-like receptors (RLRs) are a family of cytosolic DExD/H box RNA helicases involved in antiviral immunity^1^. RLRs are key sensors of pathogen-associated molecular patterns (PAMP)-containing viral RNA in infected cells^2–4^. The RLRs family includes three members: RIG-I, melanoma differentiation-associated gene 5 (MDA5) and Laboratory of Genetics and Physiology 2 (LGP2). RIG-I senses 5´-triphosphate blunt-end dsRNA, while MDA5 is activated by long dsRNA^5^. LGP2 recognizes dsRNA, regardless of 5´-triphosphate or RNA length. After viral RNA recognition, RIG-I and MDA5 interact with mitochondrial antiviral signaling protein (MAVS) through their caspase activation and recruitment domains (CARDs)^6^. Then, activation of IRF3 and NF-κB leads to the production of type-I interferon (IFN), pro-inflammatory cytokines and induction of antiviral effector genes. Unlike RIG-I and MDA5, LGP2 lacks CARDs and then independent signaling activity through MAVS. LGP2 is known to enhance MDA5-dependent IFN induction^7,8^.

The *Picornaviridae* family includes small non-enveloped positive-stranded RNA viruses of significant disease burden for humans and livestock. Among them, foot-and-mouth disease virus (FMDV), causes a highly infectious disease affecting swine, cattle and other domestic and wild animals worldwide^9,10^. Type-I IFN induction during picornavirus infections has been linked to recognition of their dsRNA replicative forms by MDA5^11–14^. When RIG-I^−/−^, MDA5^−/−^, or MAVS^−/−^ MEFs were transfected with viral RNAs of different picornaviruses an MDA5- and MAVS-dependent, IFN-β response was induced^11^. Also, LGP2 or MDA5 deficiency in mice resulted in higher susceptibility to infection by encephalomyocarditis virus (EMCV)^14,15^. However, RIG-I expression in MDA5^-/-^ MEFs conferred strong induction of IFN-β promoter early after EMCV infection^16^.

Different strategies to counteract the RLR-mediated antiviral response have been reported for picornaviruses, mainly for *Enterovirus* and *Cardiovirus*^17,18^. MDA5 was shown to be degraded during enterovirus infection in a caspase- or proteasome-dependent manner^19,20^. Later studies in infected cells support that MDA5 cleavage relies on the viral 2A protease (2Apro)^21^. Interestingly, RIG-I cleavage was also observed during infection by various enteroviruses and cardioviruses and associated with viral 3C protease (3Cpro)^16,21,22^. However, no cleavage site in MDA5 or RIG-I has been identified to date.

Little is known about cellular sensors for FMDV infection. RNA interference experiments suggested that FMDV is sensed by MDA5^23^. Also, overexpression of LGP2 can inhibit FMDV replication^24^. A differential virulence factor of FMDV is the Leader protease (Lpro), a papain-like cysteine protease with multifunctional roles on viral pathogenesis^25,26^, expressed as two forms, Lab and Lb^27^, being Lbpro more abundant in infected cells^28^. FMDV Lpro cleaves eIF4G, resulting in a rapid cap-dependent translation shut-off^29,30^. Lpro targets several cellular proteins^31^ and is involved in disruption of host defenses, degrading NF-κB p65 subunit and reducing IRF-3/7 expression^32,33^. Lpro deubiquitinase activity may affect proteins of the IFN-I signaling pathway, such as RIG-I, TBK1, TRAF3, and TRAF6^34^. Lbpro also removes ISG15 from substrate proteins by an irreversible inactivation mechanism^35^. Recently, LGP2 cleavage in the helicase motif VI by Lbpro has been reported^36^, reducing the IFN-β and antiviral response.

Little is known about the interplay between MDA5 and FMDV. Here, we demonstrate the specific role of MDA5 in sensing and impairing FMDV infection. We isolated IFN-β stimulatory RNA from MDA5 pulldowns in infected cells and report how FMDV hampers the antiviral response by targeting MDA5 for cleavage by Lpro in the conserved helicase motif VI. Remarkably, single mutations in two amino acids within the Lpro target site in MDA5 have been associated with interferonopathic diseases. Learning how viruses counteract host immune responses specifically targeting effector proteins in conserved motifs may help in designing new antiviral developments, as well as therapeutic strategies against type-I interferonopathies.

## Results

### Stimulatory FMDV RNA associates with MDA5 in infected cells

**Fig. 1:**
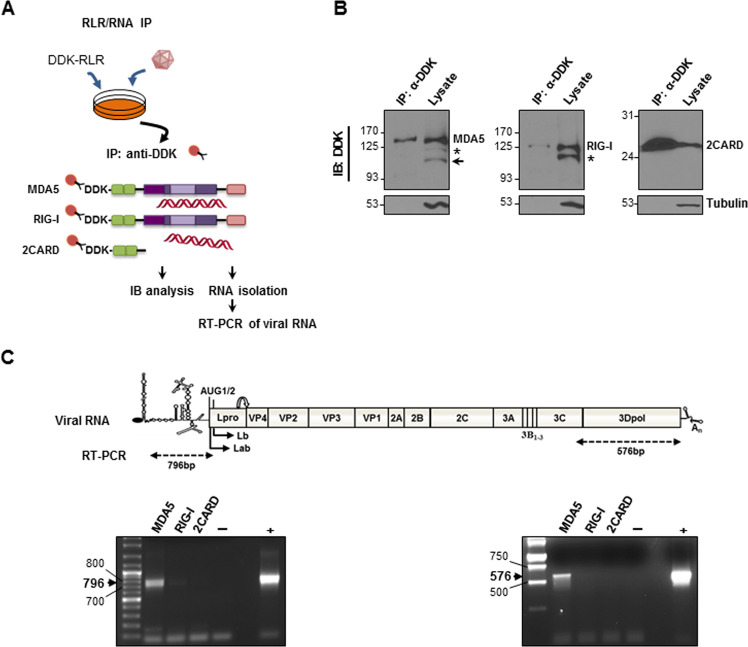
Isolation of RLR/RNA complexes in FMDV-infected cells. **a** Schematic representation of the procedure for viral RNA pulldown. **b** IBRS-2 cells were transfected with plasmids encoding DDK-tagged -MDA5, -RIG-I or -2CARD (1 µg/10^6^ cells) and infected 24 h later with FMDV O1K isolate at an MOI of 5. Cells were lysed 5 h after infection and lysates were subjected to IP and analyzed by immunoblot with an anti-DDK monoclonal antibody. A faster migration form of MDA5 (about 95 kDa) bearing the N-terminal tag is indicated with an arrow. Asterisk denotes MDA5 and RIG-I forms with slightly faster migration commonly observed in cells expressing the RLRs. **c** RNA was extracted from the IP fractions and analyzed by RT-PCR with two sets of primers for amplification of the indicated 796-bp 5´- and 576-bp 3´- terminal regions of the viral RNA. The specific IP fraction (MDA5, RIG-I or 2CARD) corresponding to each lane is indicated. Negative and positive controls were included in the RT-PCR assays (water and in vitro transcribed FMDV O1K RNA, respectively).

To find evidence of interaction between MDA5 and FMDV RNA in infected cells, tagged-RLR pulldown experiments were conducted (Fig. 1a). Swine IBRS-2 cells were transfected with DDK-tagged -MDA5 -RIG-I, or -2CARD [a RIG-I mutant lacking the helicase domain and the C-terminal domain (CTD), as nonspecific RNA-binding control] and infected 24 h later with FMDV. Cells were lysed 5 h post-infection (hpi) and MDA5/ RIG-I/, or 2CARD/RNA complexes were immunoprecipitated (Fig. 1b). In the lysates, MDA5 and RIG-I forms with slightly faster migration, commonly observed in RLRs-expressing cells, were also detected. Interestingly, a 95-kDa additional band that might correspond to an MDA5 N-terminal fragment generated during FMDV infection, was observed (arrow in Fig. 1b). Following immunoprecipitation (IP), coimmunoprecipitated RNA was isolated and analyzed by RT-PCR with primers annealing in the 5´and 3´regions of the FMDV genome (Fig. 1c). Specific amplification with both sets of primers was clearly observed for MDA5-associated RNA, unlike RIG-I or 2CARD pulldowns, indicating that viral RNA generated during FMDV infection is preferentially bound to MDA5.

**Fig. 2:**
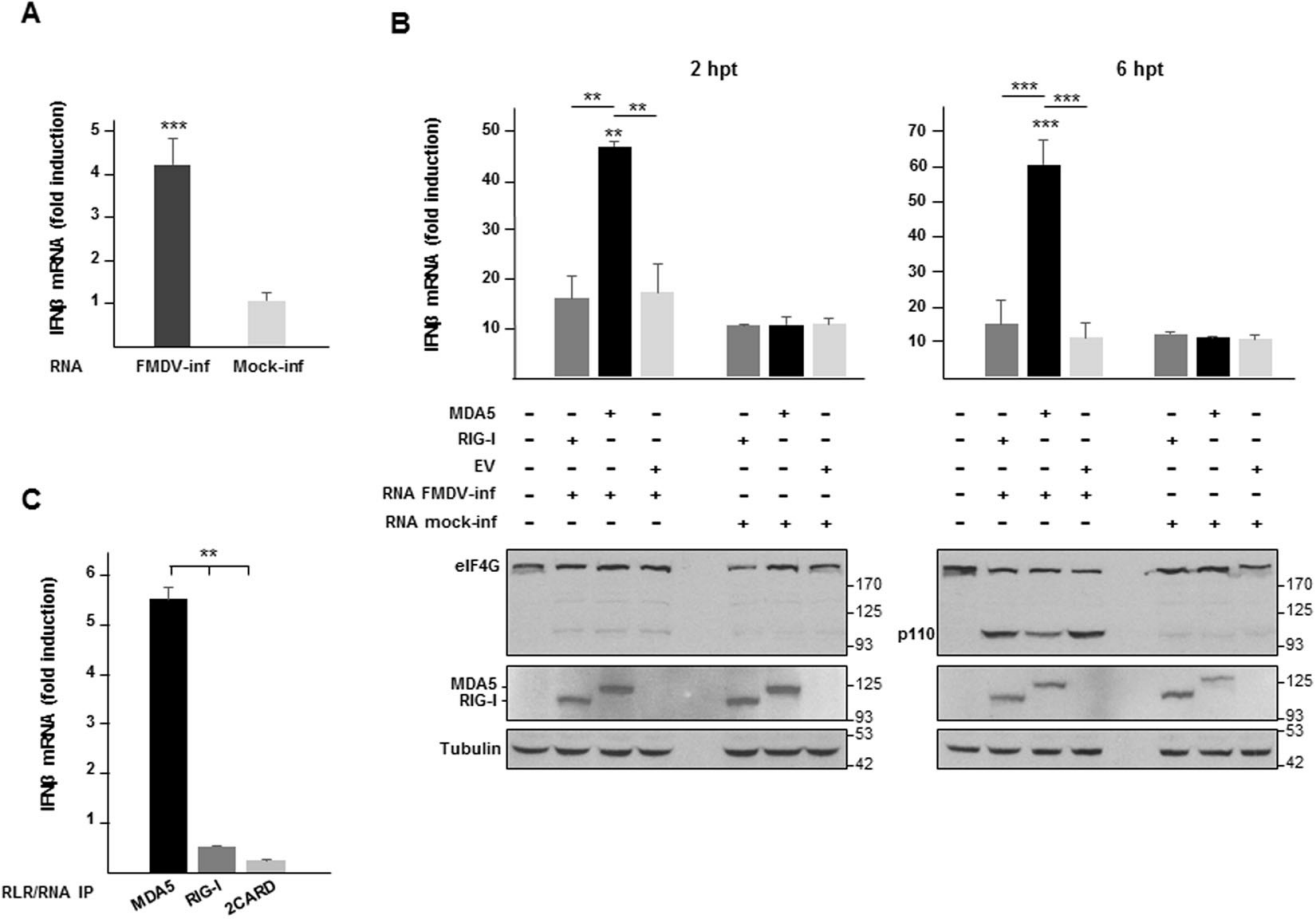
RNA derived from FMDV infection has an MDA5-dependent immunostimulatory activity. (A and B) SK6 cells were infected with FMDV O1BFS at an MOI of 5 or mock-infected and total RNA was extracted at different times after infection. **a** RNA isolated from cells 5 h after infection or mock infection was transfected into fresh SK6 cells (10 pg RNA/cell). Transfected cells were lysed 6 h later and the fold induction of porcine IFN-β mRNA in cell lysates was determined by RT-qPCR normalized to GAPDH. Data shown are mean ± SD of triplicates with comparison made to cells transfected with RNA from mock-infected cells. **b** Total RNA extracted from infected or mock-infected SK6 cells (6 µg) was used to transfect SK6 cells (5 × 10^5^) which had been transfected 24 h before with plasmids encoding DDK-tagged -MDA5, -RIG-I or -EV (1 µg/10^6^ cells). Cells were lysed 2 h or 6 hpt and the fold induction of porcine IFN-β mRNA in cell lysates was determined by RT-qPCR as in A. Data are mean ± SD of triplicates with comparisons made to cells transfected with RNA from mock-infected cells unless specified otherwise. The indicated proteins were analyzed by immunoblot. An anti-DDK monoclonal antibody was used for detection of DDK-tagged MDA5 and RIG-I. **c** SK6 cells were transfected with DDK-MDA5 and 24 h later mock-transfected or transfected with equal amounts of RNA fractions from the pulldown assay shown in Fig. 1 (1.5 µl/0.5 × 10^6^ cells) and corresponding to RNA immunoprecipitated with MDA5, RIG-I or 2CARD proteins. Cells were lysed 2 h later and the fold induction of porcine IFN-β mRNA was determined by RT-qPCR normalized to GAPDH. Data shown are mean ± SD of triplicates with comparisons made to mock-transfected MDA5-expressing cells. Significant differences using the Student´s *t* test are indicated (*p < 0.05; **p < 0.01).

To determine whether FMDV infection generates stimulatory RNA, we first measured the IFN-β mRNA levels in SK6 cells transfected with total RNA extracted from cells that had been infected for 5 h with FMDV. Significant induction of IFN-β mRNA was observed in cells transfected with RNA from FMDV-infected cells compared with those transfected with RNA from mock-infected cells (Fig. 2a). Next, we analyzed the role of MDA5 and RIG-I on the IFN induction triggered by the RNA generated during FMDV infection (Fig. 2b). Total RNA extracted from infected or mock-infected SK6 cells was used to transfect SK6 cells expressing MDA5 or RIG-I and IFN-β mRNA levels were measured at 2 or 6 h post-transfection (hpt). RNA from FMDV-infected cells only had a stimulatory effect on SK6 cells expressing MDA5 (Fig. 2b). The induction of IFN-β mRNA was higher at 6 than at 2 hpt, likely as result of viral replication. At 6 hpt eIF4G cleavage, an early event upon FMDV infection, was readily detected (p110 fragment) (Fig. 2b). To test whether the MDA5-associated viral RNA could act as a PAMP and induce an antiviral response, we analyzed the ability of the RNAs co-immunoprecipitated with MDA5, RIG-I or 2CARD to induce an MDA5-dependent IFN-β response. Only the RNA co-immunoprecipitated with MDA5 triggered an IFN-β response in SK6 cells expressing MDA5 (Fig. 2c). Altogether, our results demonstrate that immunostimulatory RNA generated during FMDV infection is primarily sensed by MDA5.

### MDA5 expression impairs FMDV replication

**Fig. 3:**
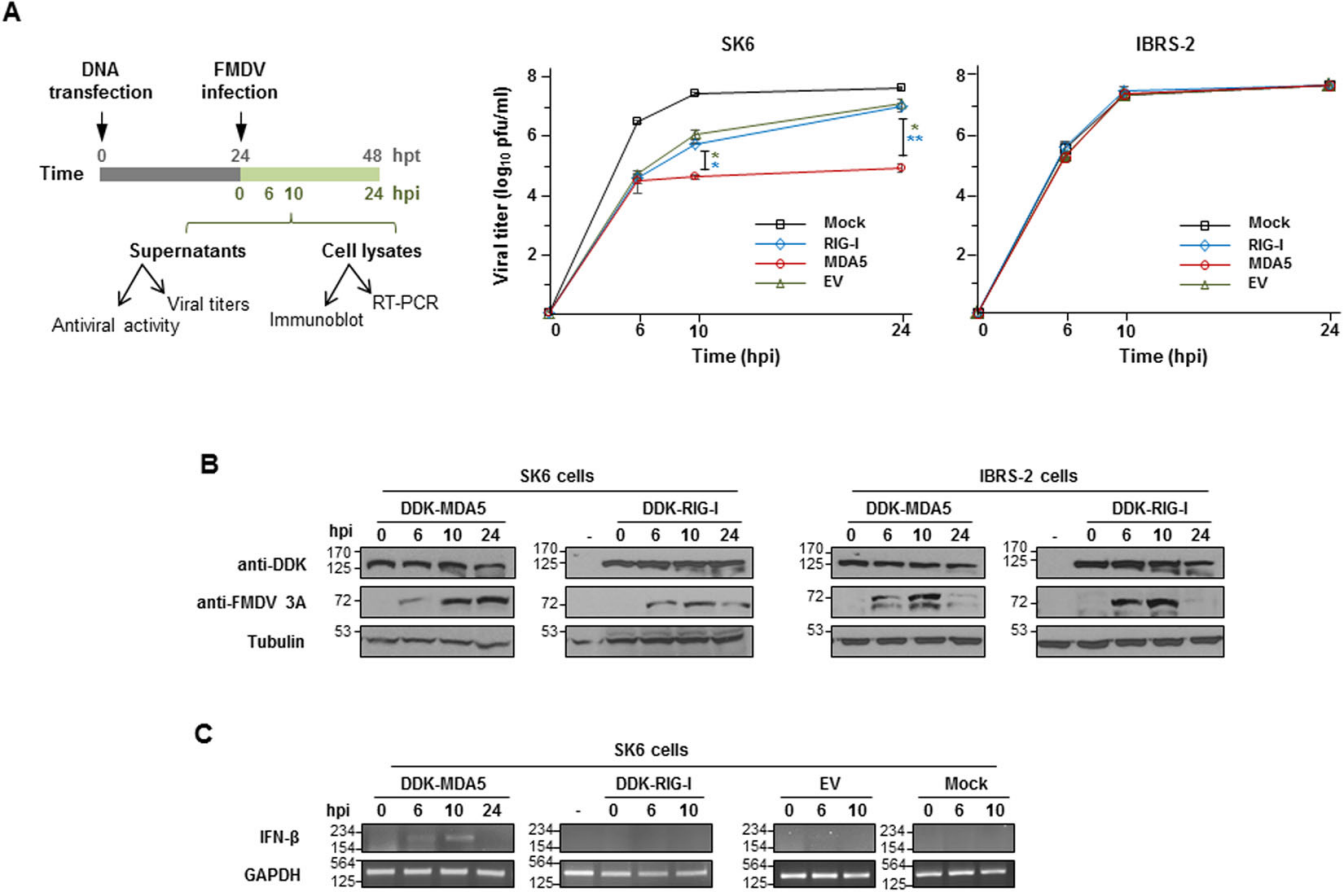
Effect of RIG-I and MDA5 expression on FMDV growth. SK6 or IBRS-2 cells (1 × 10^6^) were transfected with 2 µg of plasmids encoding DDK-MDA5, DDK-RIG-I, an empty vector (EV) or mock-transfected. Cells were infected 24 h later with FMDV O1K isolate at an MOI of 1. Supernatants were collected and cells lysed at the indicated times after infection. **a** A schematic representation of the experiment is shown. Viral titers in supernatants collected from SK6 or IBRS-2 cells were determined by plaque assay. Data are mean ± SD of duplicates of two independent experiments. Significant differences using the Student´s *t* test between viral titers in EV- or RIG-I-transfected and MDA5-transfected SK6 cells (green and blue asterisks, respectively) are indicated (*p < 0.05; **p < 0.01). **b** Cell lysates were analyzed by immunoblot for the DDK-tagged proteins and viral 3A non-structural protein. Tubulin was used for normalization. **c** RT-PCR amplification of IFN-β and GAPDH mRNAs from RNA extracted from SK6 cells transfected as specified or mock-transfected and lysed at the indicated times after infection. Due to extensive CPE at 24 hpi, RNA extraction from the few remaining attached cells was only possible for MDA5-transfected monolayers at that time point. GAPDH levels were used as loading control.

Having shown the relevance of MDA5 in sensing FMDV RNA, we analyzed the role of MDA5 and RIG-I during FMDV infection. The effect of the expression of each helicase was assayed in two different swine epithelial kidney cells; SK6 cells induce IFN in response to viral infection, while IBRS-2 cells have lost that competence. Cells were transfected with DDK-tagged RLRs or an empty vector (EV) and infected 24 h later with FMDV (MOI of 1). Expression of MDA5 in SK6 cells induced a significant reduction in viral titers compared with EV-transfected cells at 10 and 24 hpi (Fig. 3a). However, no significant differences in titers were found between RIG-I- and EV-transfected SK6 cells. Interestingly, the viral titers in IBRS-2 cells transfected with the different plasmids were similar at all time points (Fig. 3a). Expression of the RLRs and the non-structural 3A viral protein in both cell lines was confirmed by immunoblot (Fig. 3b). When the mRNA levels of porcine IFN-β were analyzed by RT-PCR in MDA5- or RIG-I-expressing SK6 cells over infection, detectable levels were only observed in MDA5-transfected cells at 6 and 10 hpi (Fig. 3c). Consistently, antiviral activity was only detected in MDA5-transfected SK6 cells at 6 and 10 hpi (Fig. S1). Altogether, our results suggest that MDA5 is the critical RLR for triggering a type-I IFN response during FMDV infection.

### MDA5 is cleaved during FMDV infection

**Fig. 4:**
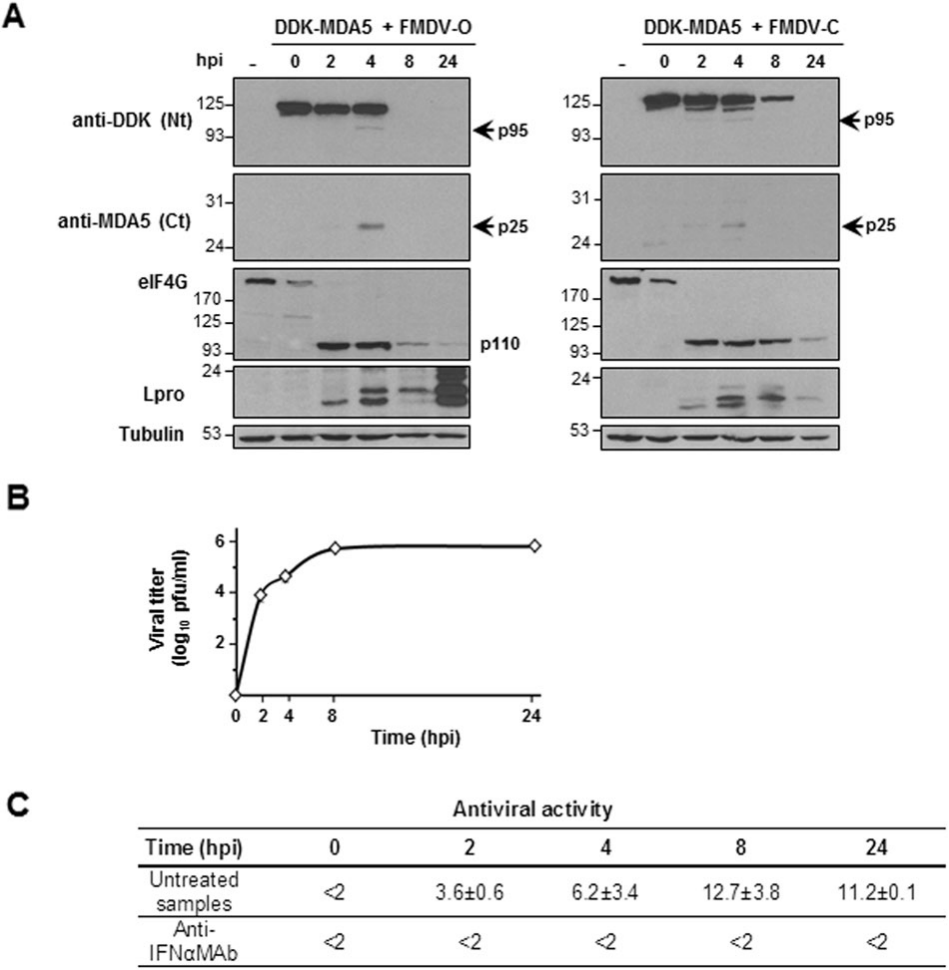
FMDV infection induces MDA5 cleavage. Swine SK6 cells were mock-transfected or transfected with 2 µg of a plasmid encoding DDK-MDA5 and 24 h later infected with type-O or type-C FMDV at an MOI of 5. Cells were lysed at different times after infection. **a** Lysates were analyzed by immunoblot for detection of the indicated proteins using the specified antibodies. Arrows indicate the N- and C-terminal cleavage products of MDA5. The 110-kDa cleavage product of eIF4G is also depicted. **b** Viral titers in supernatants collected from cells analyzed in **a** (infected with type-O virus) were determined by plaque assay on IBRS-2 cells. Data as mean of triplicates ± SD. **c** Antiviral activity in supernatants from cells analyzed in **a** (infected with type-O virus) is expressed as the reciprocal of the highest dilution of the supernatant needed to reduce the number of VSV plaques on IBRS-2 cells by 50%. When indicated, supernatants were previously treated with a monoclonal antibody anti-swine IFN-α. Data are average of three independent assays ± SD.

Proven the specific role of MDA5 on sensing FMDV infection, we investigated whether the virus counteracts the MDA5-dependent antiviral response. In the MDA5-RNA pulldown, an additional band was observed (Fig. 1b), suggesting that MDA5 might be cleaved during infection. To address that issue, MDA5-expressing SK6 cells were infected with two different-type FMDVs (MOI of 5) and the MDA5 pattern, eIF4G cleavage and Lpro expression over time were monitored by immunoblot (Fig. 4a). An inverse correlation was observed between MDA5 levels and viral Lpro accumulation, this last accounting for the extensive eIF4G cleavage observed at 2 hpi. A complete disappearance or a drastic decrease in full-length MDA5 was observed at 8 hpi together with appearance of an N-terminal product of about 95 kDa, similar to that observed in infected cells at 5 hpi (Fig. 1b). Using an antibody against the C-terminal region of MDA5, a product of about 25 kDa was detected coinciding with detection of the N-terminal product (Fig. 4a). Quantitative analysis of the 25 kDa C-terminal product in the blot revealed a 37.4 or 34.7% of cleavage at 4 hpi with type-O or type-C FMDV, respectively. In order to correlate the progression of infection with MDA5 cleavage and analyze its effect on the antiviral response, the viral titers and the antiviral activity in the supernatants over infection with the type-O virus were determined (Fig. 4b, c). The antiviral activity levels, measured as the highest dilution of supernatant inhibiting vesicular stomatitis virus (VSV) infection by 50%, increased as FMDV infection progressed, reaching maximal levels at 8 hpi (Fig. 4c). The antiviral activity was completely neutralized by incubation of the supernatants with an antibody against swine IFN-α. The highest levels of viral titers (measured by plaque assay on IBRS-2 cells) and antiviral activity overlapped with the lowest MDA5 signal (8 to 24 hpi), likely as a combination of host translation shut off and specific processing of the protein. Our results show that MDA5 is cleaved during FMDV infection in swine cells.

When the pattern of RIG-I during FMDV infection was analyzed, no direct evidence of RIG-I cleavage was observed (Fig. S2A).

### MDA5 is a target for the FMDV Leader protease

**Fig. 5:**
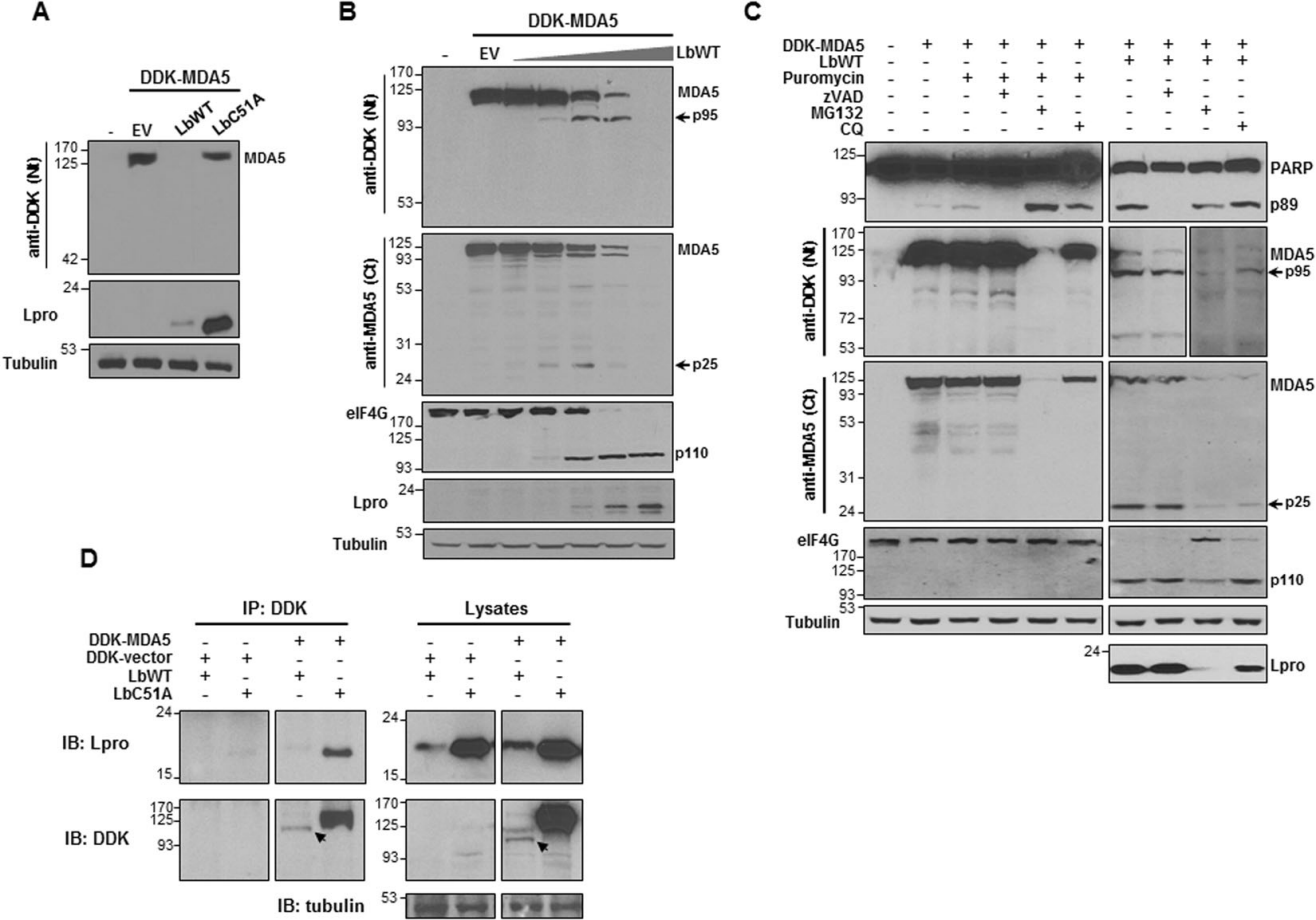
MDA5 is specifically cleaved by FMDV Lpro. HEK293 cells were mock-transfected or co-transfected with 2 µg of a plasmid encoding DDK-MDA5 or a DDK-vector and 1 µg of plasmids encoding LbWT, LbC51A or EV (**a, c** and **d**), or increasing amounts of LbWT plasmid (0.2, 2, 20, 200 and 2000 ng) or 2 µg of an EV (**b**). **c** HEK293 cells were co-transfected in the presence of zVAD (20 µM), MG132 (10 µM) or chloroquine (CQ) (50 µM). In control cells apoptosis was induced with puromycin (20 µM). Cells were lysed 24 h later and analyzed by immunoblot for detection of the indicated proteins using the specified antibodies. A longer exposure is shown for lysates from cells co-transfected in the presence of MG132 and CQ. Arrows indicate the N- and C-terminal cleavage products of MDA5. The 89-kDa and 110-kDa cleavage products of PARP and eIF4G respectively, are also depicted. **d** Lysates were collected 24 hpt and IP was performed with an anti-DDK monoclonal antibody. IP fractions and lysates were analyzed by 10%- and 8%-SDS-PAGE, respectively. The indicated proteins were analyzed by immunoblot. The N-terminal cleavage product of MDA5 is indicated with an arrow.

Next, we investigated the involvement of the virally encoded Lpro on MDA5 processing. For that, the effect of co-expression on HEK293 cells of DDK-MDA5 and either the wild type catalytically active form of Lbpro (LbWT) or an inactive form (LbC51A)^37,38^ was analyzed. When MDA5 was co-expressed with 1 µg of LbWT, the signal of the helicase was completely lost 24 hpt (Fig. 5a). Using a range of LbWT amounts, a dose effect on full-length MDA5 was observed (Fig. 5b). Interestingly, two cleavage products of about 95 and 25 kDa, corresponding to the N- and C-terminal regions of MDA5, respectively, were detected and overlapping with eIF4G cleavage (Fig. 5b).

To address whether the caspase, proteasome or lysosomal pathways were involved in the FMDV-induced MDA5 degradation, the MDA5 and LbWT co-expression assays were performed in the presence of their corresponding inhibitors zVAD, MG132 or chloroquine, respectively. Detection of the C-terminal PARP cleavage product (89 kDa) was monitored as a marker of apoptosis. As shown in Fig. 5c, the MDA5 cleavage products were generated in the presence of the inhibitors with similar pattern to the one observed in non-treated cells. On the other hand, induction of apoptosis with puromycin did not result in MDA5 cleavage. These results suggest that MDA5 degradation was specifically attributable to the protease activity of Lbpro.

When the RIG-I pattern in co-expression with Lbpro was analyzed, no evidence of cleavage was detected. Despite the protein signal loss 24 hpt with 1 µg of LbWT (Fig. S2B), likely due to Lpro-induced translation inhibition, no RIG-I degradation products were detected with increasing amounts of LbWT (Fig. S2C).

We further investigated whether MDA5 and Lbpro interact within cells (Fig. 5d). LbC51A co-immunoprecipitated very efficiently with MDA5, while LbWT was detected as a faint band. LbC51A is expressed more efficiently than LbWT and accumulates in transfected cells^36^. The amount of full-length MDA5 24 hpt with LbWT was very low, being only detectable the N-terminal cleavage product, suggesting that LbWT is still able to bind that MDA5 fragment. Altogether, these results show that FMDV induces MDA5 cleavage via Lpro activity.

### The Leader protease cleaves MDA5 at a conserved helicase motif

**Fig. 6:**
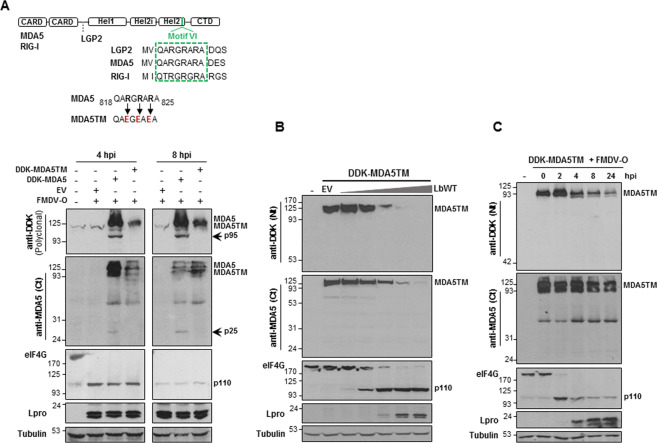
MDA5TM is resistant to Lbpro cleavage during FMDV infection. **a** A schematic representation of the three RLRs and the amino acid sequence of helicase motif VI, including residues modified in MDA5TM (R820E, R822E, R824E), are shown. SK6 cells were mock-transfected or transfected with 2 µg of DDK-MDA5, DDK-MDA5TM plasmids or an EV, then infected 24 h later with type-O FMDV at an MOI of 5 and lysed 4 or 8 h after infection. Lysates were analyzed by immunoblot for detection of the indicated proteins. In this case, a polyclonal anti-DDK antibody was used for detection of DDK-tagged MDA5 and MDA5TM. Arrows indicate the N- and C-terminal cleavage products of MDA5. The 110-kDa cleavage product of eIF4G is also depicted. **b** HEK293 cells were mock-transfected or co-transfected with 2 µg of a plasmid encoding DDK-MDA5TM and increasing amounts of LbWT plasmid (0.2, 2, 20, 200 and 2000 ng) or 2 µg of an EV. Cells were lysed 24 h later and analyzed by immunoblot for detection of the indicated proteins. **c** Swine SK6 cells were mock-transfected or transfected with 2 µg of a plasmid encoding DDK-MDA5TM and 24 h later infected with type-O FMDV at an MOI of 5. Cells were lysed at different times after infection and analyzed by immunoblot for detection of the indicated proteins as in **b**.

As the Lpro target site in LGP2 is present in MDA5 (Fig. 6a) and the cleavage pattern was consistent with Lpro cleaving in the same region, MDA5TM mutant was generated. In MDA5TM the stretch of positively charged R amino acids RGRAR was changed to negatively charged glutamic acid (EGEAE) (Fig. 6a). An equivalent triple substitution abolished LGP2 cleavage by Lbpro^36^. SK6 cells were transfected with DDK-MDA5 or DDK-MDA5TM plasmids, infected with type-O FMDV (MOI of 5) and lysed 4 or 8 hpi. While both the 95 kDa N-terminal and the 25 kDa C-terminal MDA5 cleavage products were clearly detected at 4 and 8 hpi, no MDA5TM degradation products were observed (Fig. 6a). Similarly, the lack of MDA5TM cleavage was further confirmed by co-expression with increasing amounts of LbWT (Fig. 6b) and in the context of FMDV infection up to 24 hpi (Fig. 6c). Nevertheless, the impact on the overexpressed MDA5TM cap-dependent translation of either increasing amounts of LbWT (Fig. 6b) or the virally encoded Lpro over infection (Fig. 6c), both associated with eIF4G cleavage, was evident. Our results show that Lpro cleaves MDA5 at the RGRAR motif, suggesting that FMDV is targeting the two key viral sensors involved in the antiviral response against picornavirus infection, MDA5 and LGP2, at the same conserved domain. To our knowledge, this is the first report of the specific cleavage site on MDA5 of a viral protease.

### MDA5 cleavage by Lpro impacts negatively on the antiviral response

**Fig. 7:**
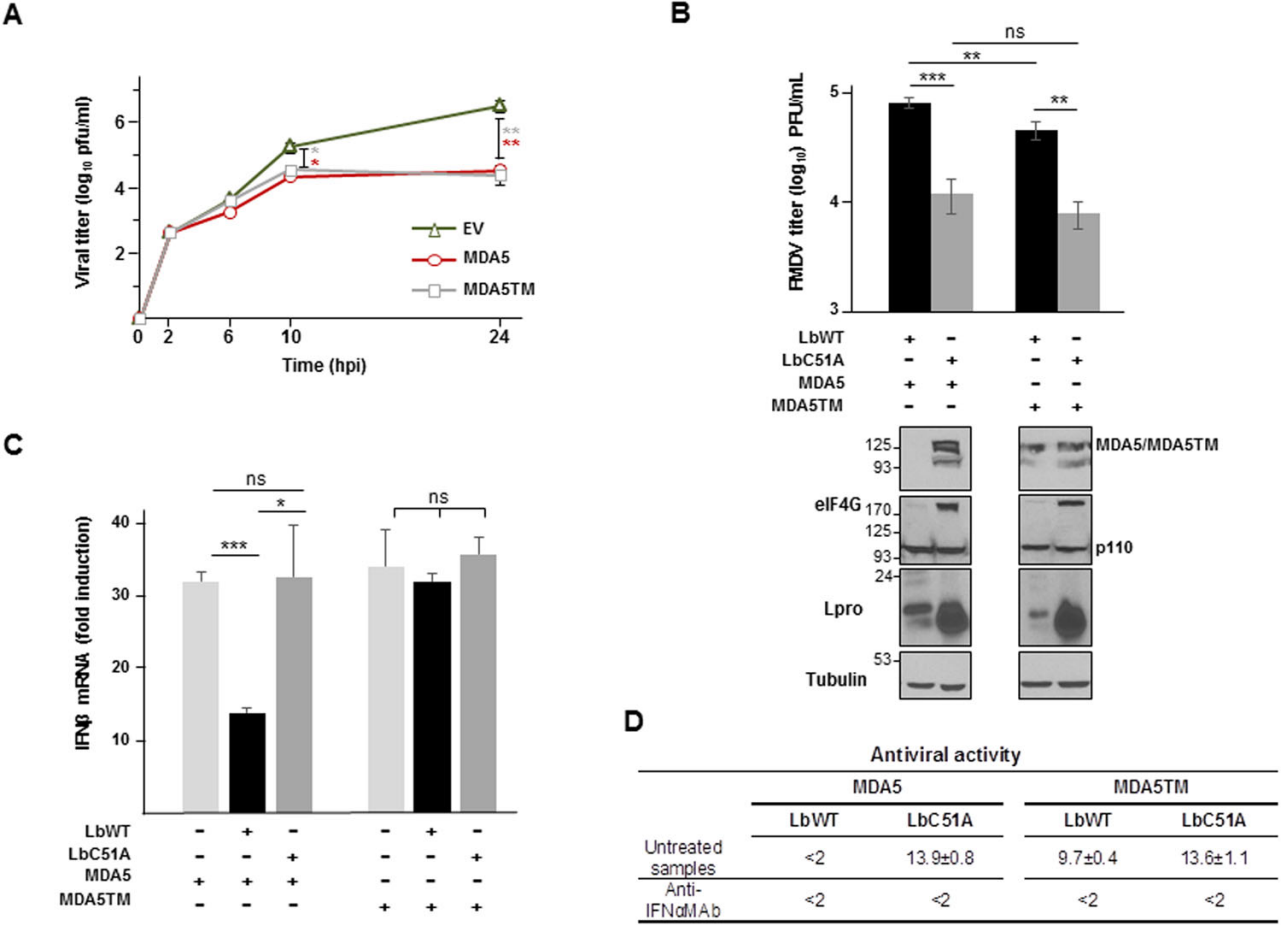
Effect of MDA5 cleavage on the antiviral response in swine cells. **a** SK6 cells (1 × 10^6^) were transfected with 2 µg of a plasmid encoding DDK-MDA5, DDK-MDA5TM or an EV and infected 24 h later with FMDV O1K isolate at an MOI of 1. Supernatants were collected at the indicated times after infection and viral titers were determined by plaque assay. Data are average of triplicates ± SD. Significant differences using the Student´s *t* test between viral titers in MDA5- or MDA5TM-transfected and EV-transfected SK6 cells (black and gray asterisks, respectively) are indicated (*p < 0.05; **p < 0.01). **b**–**d** SK6 cells (1 × 10^6^) were co-transfected with the indicated plasmids (2 µg of DDK-MDA5 or MDA5TM; 1 µg of LbWT or LbC51A) and infected 24 h later with FMDV Cs8 isolate at an MOI of 5. Supernatants were collected and cells were lysed 8 h after infection. **b** Viral titers in the supernatants were determined by plaque assay. Data are mean ± SD of triplicates. Lysates were analyzed by immunoblot for the indicated proteins; MDA5 and MDA5TM were detected with an anti-MDA5 (C-terminal) polyclonal antibody; the 110-kDa cleavage product of eIF4G is depicted. **c** The fold induction of porcine IFN-β mRNA in cell lysates was determined by RT-qPCR normalized to GAPDH. Data are mean ± SD of triplicates. Significant differences using the Student´s *t* test are indicated (*p < 0.05; **p < 0.01; ns not significant). **d** Antiviral activity in the supernatants was measured by IFN bioassay and is expressed as the reciprocal of the highest dilution needed to reduce the number of VSV plaques on IBRS-2 cells by 50%. When indicated, supernatants were previously treated with a monoclonal antibody anti-swine IFN-α. Data are average of duplicates ± SD.

We further asked whether MDA5 cleavage by Lpro was involved in counteracting the host antiviral response. For that, we took advantage of the non-cleavable MDA5 version of the protein (MDA5TM), which interestingly, preserved full antiviral activity against FMDV infection (Fig. 7a). When MDA5 or MDA5TM were expressed in SK6 cells, no significant differences were observed between the viral titers recovered at several times after FMDV infection, both more than 2-log lower than EV-transfected control. Next, we analyzed the viral titers, the IFN-β mRNA levels, and the antiviral activity in SK6 cells co-expressing MDA5 or MDA5TM and either LbWT or LbC51A at 8 hpi with FMDV (MOI of 5). A significant reduction in FMDV titers was observed when LbWT was co-expressed with MDA5TM compared with MDA5 (1.76-fold, *p* = 0.007), while no relevant differences were observed in co-expression with LbC51A (Fig. 7b). In contrast, viral titers in cells expressing LbC51A and either MDA5 or MDA5TM were reduced compared with those in cells expressing catalytically active Lb (compare the corresponding black and gray bars). These results are consistent with the complete cleavage of eIF4G observed when active Lbpro is expressed (Fig. 7b). In cells co-expressing MDA5 and LbWT, the helicase was not detected, likely due to the combined activity of both the FMDV-encoded Lpro and overexpressed LbWT. Consistently, expression of LbWT induced a drastic reduction in IFN-β mRNA levels in cells expressing MDA5 but not in those expressing MDA5TM. No significant decrease in IFN-β mRNA was induced by co-expression of LbC51A with MDA5 or MDA5TM (Fig. 7c). Although similar levels of antiviral activity were detected in cells co-expressing LbC51A and either MDA5 or MDA5TM (Fig. 7d), no antiviral activity was found in supernatants of cells co-expressing MDA5 and LbWT. However, the antiviral activity measured in cells co-transfected with LbWT and MDA5TM was only slightly lower than in cells expressing LbC51A (Fig. 7d), showing that despite the Lpro-induced translation shut-off, a significant level of IFN-dependent antiviral activity induced by non-cleavable MDA5TM remains in those cells. On the contrary, MDA5 cleavage by Lbpro leads to a complete loss of the antiviral activity.

**Fig. 8:**
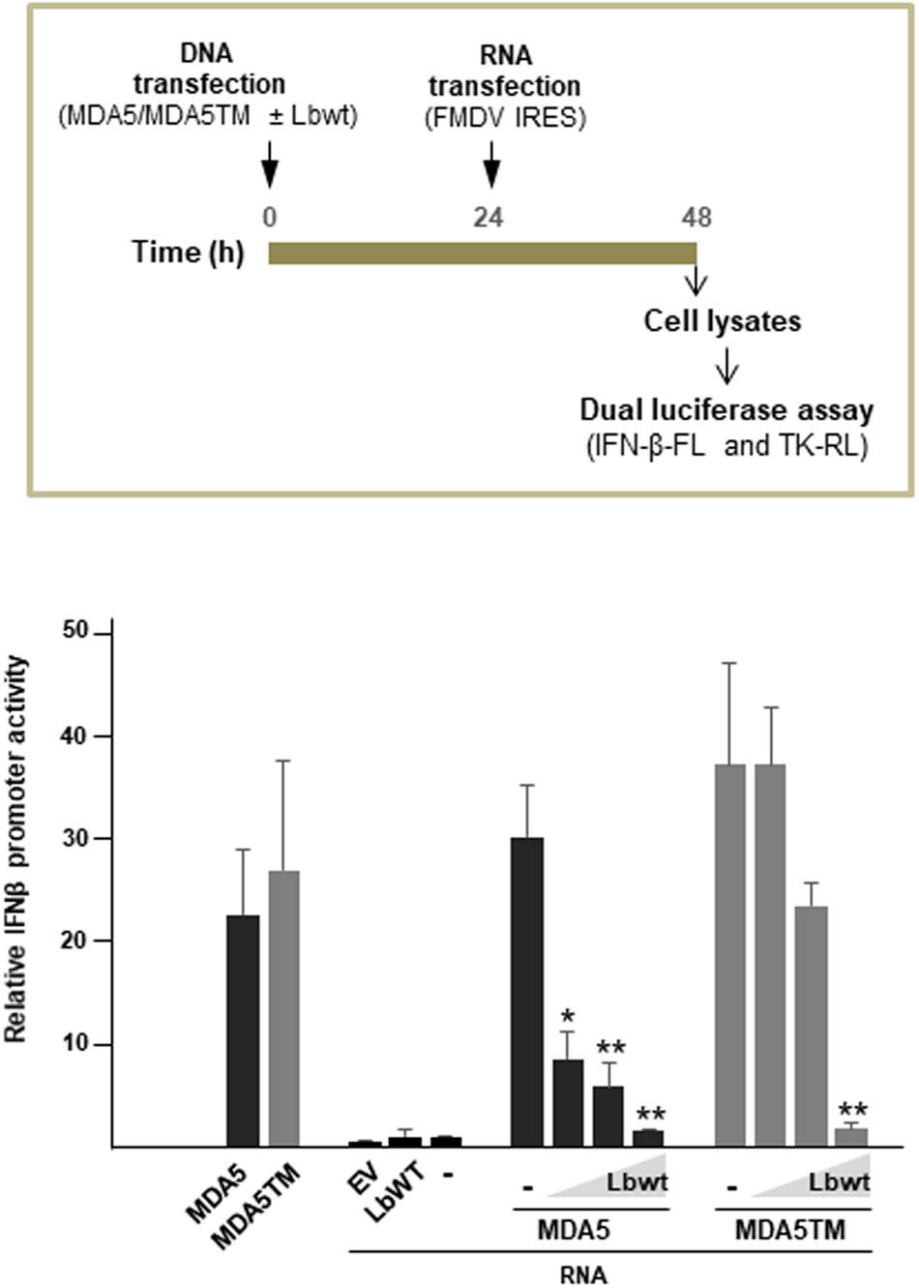
Dose effect of Lbpro on MDA5-dependent IFN-β induction. HEK293 cells (1 × 10^6^) were co-transfected with 50 ng of pIFN-β-FL, 25 ng of pRL-TK, and 10 ng of DDK-MDA5 or DDK-MDA5TM and increasing amounts of LbWT plasmid (0.2, 2 and 20 ng). Cells were also co-transfected with an EV or LbWT plasmid (20 ng) as controls. After 24 h, cells were mock-treated or stimulated with FMDV IRES RNA transcripts. Cells were harvested 24 h later and IFN-β induction was measured by dual-luciferase assay detecting expression of firefly luciferase under the human IFN-β promoter normalized to renilla activity. Data are average of duplicates of two independent assays ± SD. Significant differences using the Student´s *t* test are indicated with comparisons made to RNA-treated MDA5 or MDA5TM cells (LbWT-) (*p < 0.05; **p < 0.01).

We further confirmed the detrimental effect of MDA5 cleavage by Lbpro on the IFN-β response using a synthetic RNA derived from the FMDV genome (IRES), known to stimulate MDA5^39^ (Fig. 8). Co-transfection of HEK293 cells with MDA5 and different amounts of LbWT induced a rapid and efficient reduction in the IFN-β promoter activity, while its effect in co-expression with MDA5TM was milder, requiring a higher dose of LbWT to impair IFN-β induction. In both cases, as expected, high levels of expression of LbWT lead to severe impairment of IFN-β induction.

Taken together, our results show that specific cleavage of MDA5 by Lpro dampens the antiviral response triggered by the helicase in response to viral infection or transfection with a PAMP RNA.

## Discussion

Antiviral response against picornaviruses seems to rely on detection by MDA5 of the dsRNA intermediates generated during infection. However, RIG-I degradation, and RIG-I-dependent IFN-β induction in MDA5^-/-^ MEFs during enteroviruses infection have been reported^16,21,22^. Here, we unveil the intricate interplay between FMDV and MDA5 providing direct evidence of the MDA5 role in sensing and impairing FMDV infection. We isolated stimulatory viral RNA by MDA5 pulldown in FMDV-infected swine cells and MDA5 expression induced a strong IFN-dependent inhibitory effect on FMDV infection. Neither inhibition of viral growth was detected in cells expressing RIG-I nor viral RNA could be amplified from RIG-I pulldowns.

Interestingly, we found evidence of MDA5 cleavage by Lpro during FMDV infection. Lpro is a powerful virulence factor reinforcing the ability of FMDV to neutralize both, the host innate and adaptive immune response, at many levels^25,40^. Unfortunately, we were unable to detect cleavage of endogenous MDA5 due to the low levels of expression of the protein and the limited cross-reactivity of the antibodies available in swine cells. Our attempts to induce MDA5 expression by treatment of cells with recombinant porcine IFN-α or RNA transfection prior to infection resulted in detectable levels of the protein but also in a complete protection against infection by FMDV, known to be extremely sensitive to type-I IFN.

Degradation of MDA5 and RIG-I does not seem to be common to all picornaviruses^17^. Moreover, no specific cleavage site has been identified to date on MDA5 or RIG-I. Some reports have described the caspase- or proteasome-mediated MDA5 degradation during enterovirus infection^19,20^. The MDA5 cleavage pattern observed during FMDV infection or Lbpro expression was independent of activation of cellular apoptosis, proteasome or lysosomal pathways. RIG-I cleavage by EMCV has been linked to 3Cpro activity^22^ or to a combination of 3Cpro and caspase activities^16^. However, Feng et al. reported that MDA5 and RIG-I remained intact during EMCV infection while MDA5 degradation during enteroviruses infection was associated with 2Apro expression^21^. We did not detect any RIG-I-derived degradation products during FMDV infection or Lbpro expression.

We previously reported LGP2 cleavage by FMDV Lpro at the RGRAR sequence within motif VI in helicase domain 2 (Hel2)^36^. That sequence is present in MDA5 and other RNA-binding proteins^41^. A mutant protein (MDA5TM) bearing three substitutions known to abolished LGP2 cleavage by Lbpro^36^, proved to be cleavage resistant, identifying MDA5 as a new cellular target for Lpro and the RGRAR motif as the specific cleavage site. Interestingly, slight differences in the amino acid sequence of RIG-I motif VI compared to MDA5 or LGP2 were observed.

As the non-cleavable MDA5TM retained full antiviral activity, we could clearly link MDA5 cleavage by Lbpro, leading to the excision of the 25 kDa C-terminal fragment, to dampening of the antiviral response against FMDV infection in swine cells. Previous work showed that deletion of motif VI resulted in inactive MDA5 for antiviral signaling^42^. The CTD is also important for efficient dsRNA recognition through its close collaboration with the helicase domain providing RNA specificity^43,44^. Small amounts of LGP2 have been reported to enhance MDA5-mediated signaling^7,45^. This enhancing effect requires conserved RNA-binding determinants in both the CTD and helicase^46^. Additionally, an RNA derived from EMCV infection with MDA5-stimulatory activity was identified through its interaction with LGP2^47^. Lpro cleavage at the RGRAR sequence would remove part of the helicase motif VI and the CTD from MDA5 and LGP2, yielding inactive proteins for antiviral signaling. These findings suggests that FMDV has developed an “all-in-one” solution to prevent the antiviral response induced by both proteins individually as well as synergistically, giving the virus a clear advantage for replication and spread in the host.

Positive and negative regulatory mechanisms of RIG-I and MDA5 are necessary to tightly control IFN signaling. Over activation of MDA5 has been associated with autoimmune disorders including Singleton-Merten syndrome (SMS), Aicardi–Goutières syndrome (AGS) and systemic lupus erythematosus (SLE)^48,49^. A gain-of-function missense mutation R822Q in MDA5-coding gene *IFIH1* leading to aberrant and continuous signaling of type-I IFNs and inflammatory cytokines, has been identified in SMS and SLE patients^50,51^. Mutant mice harboring a single substitution in G821, next to R822 in MDA5, developed lupus-like nephritis and systemic autoimmune symptoms^49,52^. Both R822 and G821 are located within Hel2 motif VI of MDA5. This site, overlapping with the Lpro target site, has been suggested as a “hot spot” for causing bone abnormalities in mice and humans^49^. Although a slight increase in IFN-β induction during FMDV infection was observed in cells expressing MDA5TM (bearing mutation R822E) compared with MDA5 (Figs. 7c and 8), no statistically significant differences were observed between them, and the antiviral activity measured in the corresponding supernatants was very similar (Fig. 7d). Thus, current data do not support a correlation between the IFN levels induced by MDA5TM relative to MDA5 and the interferonopathies associated with mutations in the helicase motif VI. As no definitive therapeutic treatment has been established for patients with interferonopathic diseases, the use of viral IFN suppressors to subdue the activity of hyperactive signaling proteins is starting to be explored. Paramyxovirus V proteins interact with MDA5 and LGP2 abolishing MDA5-dependent signaling^53,54^ and its co-activation by LGP2^55^. These proteins have been shown to disrupt chronic signaling and antiviral activity of MDA5 proteins with mutations that confer hyperactive signaling profiles in patients with interferonopathic disorders^56^.

In-depth understanding of the mechanistic of innate immunity seems crucial for development of novel therapies to combat microbial infection. Also, lessons learned from viral pathogens may help in the treatment and prevention of diseases that involve the immune system.

## Materials and methods

### Cells and viruses

HEK293 (ATTC), SK6 and IBRS-2 (CISA-INIA) cells were grown in DMEM (GIBCO), 10% FBS, 2 mM glutamine and penicillin/streptomycin (100 U/ml) at 37 °C with 5% CO_2_. FMDV type-C CS8, type-O O1BFS and O1K and VSV were propagated in SK6 or IBRS-2 cells. Viral titers were determined by plaque assay^36^ and expressed as plaque-forming unit (pfu)/ml.

### Plasmids and transfection

Plasmids encoding FMDV LbWT or LbC51A have been described^36^. Plasmids encoding DDK-tagged human MDA5 and RIG-I and pIFN-β-FL were provided by Adolfo García-Sastre. MDA5TM (R820E, R822E, R824E) was generated by site-directed mutagenesis (NZYMutagenesis, nzytech). Unless otherwise specified, 2 µg of MDA5-, MDA5TM-, RIG-I- or 2CARD-encoding plasmids and 1 µg of LbWT or LbC51A plasmids were used to transfect 10^6^ cells. DNA was balanced to 3 µg with EV. In some experiments, medium was supplemented with 20 μM Puromycin (Sigma-Aldrich), 20 μM zVAD-FMK (Promega), 10 μM MG132 (Cayman Chemical) or 50 µM chloroquine (Sigma-Aldrich). Total RNA extracted from FMDV-infected cells with TriReagent (Sigma), RNA from RLR-pulldowns or IRES transcripts^57^ were also transfected with Lipofectamine 2000 (Invitrogen).

### Immunoblotting

Protein extraction and immunoblot were performed as described^36^ using primary antibodies against MDA5 (C-16, against a C-terminal peptide of hMDA5), RIG-I (C-15, against a C-terminal peptide of hRIG-I), eIF4G (D-20, Santa Cruz), anti-FLAG (M2 and F7425, Sigma-Aldrich), anti-FMDV 3 A (2C2, Emiliana Brocchi^58^), anti-cleaved PARP (19F4, Cell Signaling), anti-FMDV Lpro (against Lab/Lb fusion protein^59^, Ewald Beck) and anti-βII-tubulin^60^. Horseradish peroxidase-conjugated goat anti-rabbit, rabbit anti-goat (Invitrogen) or anti-mouse (Thermo Fisher Scientific) IgG were used for detection of membrane-bound proteins. Band intensity was quantified in some cases by ImageJ software.

### RNA/protein complex immunoprecipitation

IBRS-2 cells were transfected with DDK-tagged -MDA5, -RIG-I or -2CARD plasmids (1 µg/10^6^ cells), infected 24 h later with FMDV O1K (MOI of 5) and lysed 5 hpi. Lysates were subjected to overnight preclearing with protein G-agarose (Roche) at 4 °C and IP with anti-DDK monoclonal antibody (M2, Sigma-Aldrich). Agarose beads were washed 3 times in ice-cold NT2 buffer (0.05% NP-40, 50 mM Tris-HCl pH 7.4, 150 mM NaCl, 1 mM MgCl_2_), resuspended in DEPC-treated water and heated at 55 °C for 15 min. RNA was isolated by phenol/chloroform extraction and ethanol precipitation with 1 µg of *E. coli* MRE600 tRNA (Roche) and resuspended in 10 µl of DEPC-treated water.

### RT-PCR and qPCR analysis

RNA extracted from IP fractions (1 µl) was analyzed by RT-PCR with two primers sets as follows: 48 °C for 30 min and 95 °C for 10 min; 35 cycles of 94 °C for 30 s, 55 °C for 45 s and 72 °C for 50 s, and then 72 °C for 7 min. Amplification with SB3 (5´-TAAGTTTTACCGTCTGTCCCGAC-3´) and ATG2 (5´-CAGTTCCATTTTCCCTGTGGTGCG-3´) yields a 796-bp product enclosing the pseudoknots and the IRES regions up to the second initiator AUG. Amplification with 3DNEST (5´-GGTCCATGCTTCTTAAAATGAAGG-3´) and SB10 (5´- GGCACGTACTTTCTCCATCGGGCG-3´) yields a 576-bp fragment of the 3C-3D region. RNA transcripts (10 pg) from FMDV O1K full-length clone and water were used as positive and negative controls, respectively. Aliquots were analyzed on 1% agarose gels. Porcine IFN-β and GAPDH primers have been described^39^.

Quantitative analysis of swine IFN-β gene expression was analyzed by RT-qPCR. Total RNA (500 ng) isolated with TriReagent (Sigma) and DNase-treated (Turbo DNA-free, Ambion) was used for RT with 20 U of SuperScript III RT (Invitrogen) at 55 °C for 30 min. RT aliquots (1/10) were used for qPCR with SYBR green I (Roche). Relative expression was calculated using the ΔΔ*C*_*T*_ method normalizing to GAPDH. IFN-β and GAPDH primers were previously described^57^.

### Antiviral activity

Antiviral activity was determined by VSV infection inhibition on IBRS-2 cells^57^. Briefly, SK6 cells were plasmid-transfected and 24 hpt infected with FMDV (MOI of 5). Supernatants were collected 7 hpi and infectious particles inactivated by acidic treatment followed by neutralization. IBRS-2 monolayers were incubated with supernatants for 24 h at 37 °C, washed and infected with VSV (50–60 pfu/1 × 10^6^ cells). Antiviral activity was defined as the highest supernatant dilution inducing a 50% reduction of plaques 24 hpi. In some assays, supernatants were incubated for 1 h at 37 °C with 1 µg of anti-swine IFN-Alpha antibody (K9, PBL).

### MDA5/Lpro co-immunoprecipitation

HEK293 cells were co-transfected with 2 µg of DDK-MDA5 or a DDK-vector and 1 µg of LbWT or LbC51A plasmids. Lysates were harvested 24 hpt in 100 µl of lysis buffer (50 mM Tris-HCl [pH, 7.5], 150 mM NaCl, 0.5% NP-40, and protease inhibitor cocktail) centrifugated at 10,000 x *g* for 5 min at 4 °C and precleared with 25 µl of protein G-Agarose (Roche) for 1 h at 4 °C. Then, IP was performed with anti-DDK antibody (M2, Sigma-Aldrich)^36^. IP fractions and lysates were analyzed by 8–10%-SDS-PAGE and immunoblot.

### IFN reporter assays

HEK293 cells (1 × 10^6^) were co-transfected with 50 ng of a reporter plasmid expressing firefly luciferase under the hIFN-β promoter (pIFN-β-FL), 25 ng of renilla luciferase reporter plasmid (pRL-TK) (Promega), 10 ng of DDK-MDA5 or DDK-MDA5TM and increasing amounts of LbWT plasmid (0.2–20 ng). EV or LbWT plasmids (20 ng) were co-transfected as controls. After 24 h, cells were mock-treated or stimulated with FMDV IRES transcripts (0.2 µg/ml). Cells were harvested 24 hpt and IFN-β induction measured (Dual-Luciferase Reporter Assay, Promega). Firefly luciferase activity was normalized to renilla and expressed as fold differences relative to mock-transfected cells.

### Statistical analysis

The unpaired Student’s *t* test for independent samples was used to compare data using IBM SPSS software; statistically significant differences are indicated with asterisks in the figures (***p < 0.001, **p < 0.01, *p < 0.05); ns not significant (*p* > 0.05).

## Supplementary information

Fig S1. Antiviral activity in RLR-transfected and FMDV-infected swine cells.

Supplemental Fig 1 legend

Fig S2. Analysis of RIG-I pattern during FMDV infection or in co-expression with Lbpro.

Supplemental Figure 2 legend
