# Supplementary figures and images for: MDA5 cleavage by the Leader protease of foot-and-mouth disease virus reveals its pleiotropic effect against the host antiviral response
Source: Cell Death Dis. 2020 Sep 2;11(8):718. doi: 10.1038/s41419-020-02931-x (PMC7468288; doi:10.1038/s41419-020-02931-x)

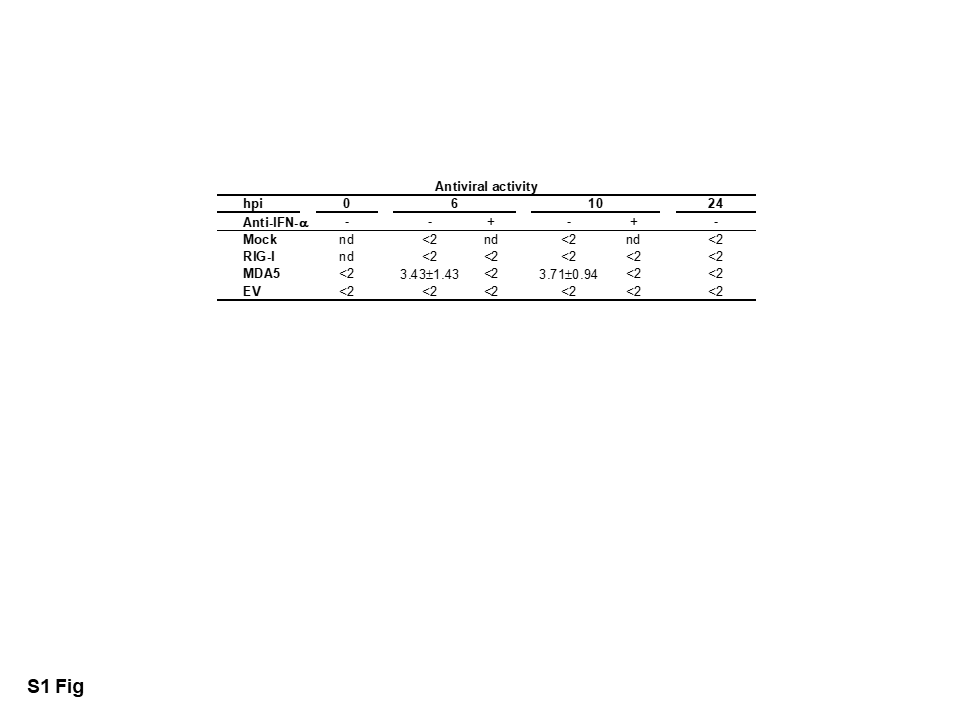

Supplement: Supplementary file 1 — Fig S1. Antiviral activity in RLR-transfected and FMDV-infected swine cells. [file 41419_2020_2931_MOESM1_ESM.tif]

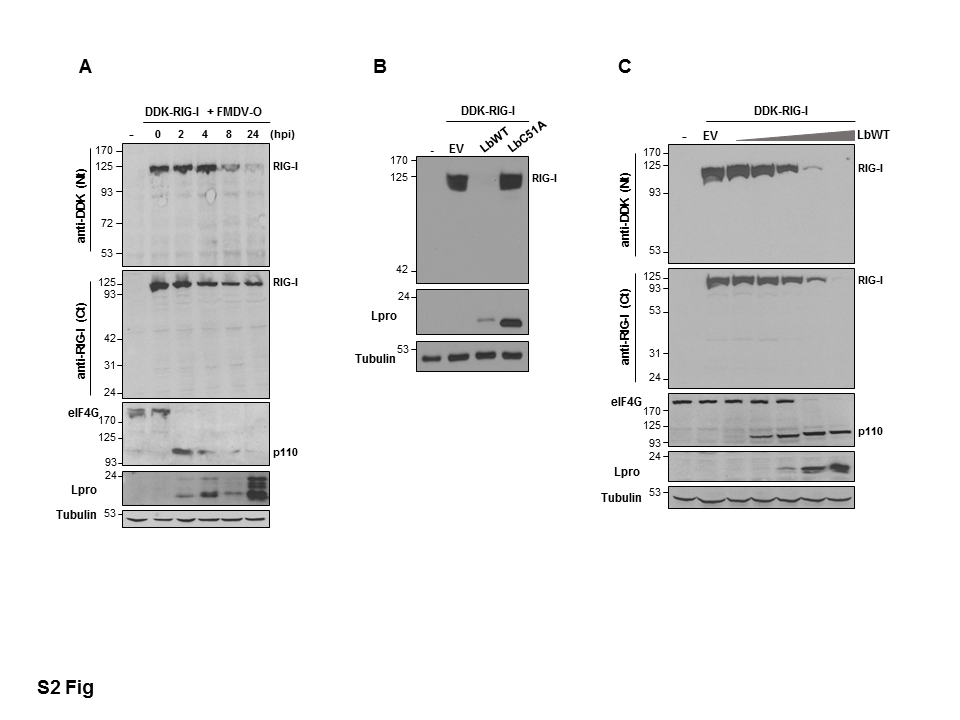

Supplement: Supplementary file 3 — Fig S2. Analysis of RIG-I pattern during FMDV infection or in co-expression with Lbpro. [file 41419_2020_2931_MOESM3_ESM.tif]
