# Supplementary material for: MDA5 cleavage by the Leader protease of foot-and-mouth disease virus reveals its pleiotropic effect against the host antiviral response
Source: Cell Death Dis. 2020 Sep 2;11(8):718. doi: 10.1038/s41419-020-02931-x (PMC7468288; doi:10.1038/s41419-020-02931-x)
Supplement: Supplementary file 2 — Supplemental Fig 1 legend [file 41419_2020_2931_MOESM2_ESM.docx]

**Fig S1. Antiviral activity in RLR-transfected and FMDV-infected swine cells.** Antiviral activity in the supernatants corresponding to Fig 3 was measured by IFN bioassay and is expressed as the reciprocal of the highest dilution needed to reduce the number of VSV plaques on IBRS-2 cells by 50%. When indicated, supernatants were previously treated with a monoclonal antibody anti-swine IFN-α. Data are average of duplicates ± SD; nd, not done.
