# Supplementary material for: MDA5 cleavage by the Leader protease of foot-and-mouth disease virus reveals its pleiotropic effect against the host antiviral response
Source: Cell Death Dis. 2020 Sep 2;11(8):718. doi: 10.1038/s41419-020-02931-x (PMC7468288; doi:10.1038/s41419-020-02931-x)
Supplement: Supplementary file 4 — Supplemental Figure 2 legend [file 41419_2020_2931_MOESM4_ESM.docx]

**Fig S2. Analysis of RIG-I pattern during FMDV infection or in co-expression with Lbpro.** (A) SK6 cells were mock-transfected or transfected with 2 µg of a plasmid encoding DDK-RIG-I and 24 h later infected with type-O FMDV at an MOI of 5. Cells were lysed at the indicated times after infection. (B and C) HEK293 cells were mock-transfected, co-transfected with a plasmid encoding DDK-RIG-I (2 µg) and plasmids encoding LbWT or LbC51A (1 µg) (B), increasing amounts of a plasmid encoding LbWT (0.2, 2, 20, 200 and 2000 ng) (C) or an EV (2 µg). Cells were lysed 24 h later. Lysates were analyzed by immunoblot for detection of the indicated proteins using the specified antibodies. The 110-kDa cleavage product of eIF4G is also depicted.
